# Monthly Summary

**Published:** 1876-07

**Authors:** 


### MONTHLY SUMMARY.

---

*A More Effectual Mode of Applying Iodine to the Interior of Certain Cysts.*—Mr. Furneux Jordan, in some notes in the *Lancet*, remarks that he has found in practice two classes of scrotal hydroceles, in which the ordinary methods of treatment are either difficult to use or uncertain in their result. In boys and men there are occasionally encysted hydroceles of the testis, or the cord, which continue to increase in size, or in which treatment is urgently requested. In such cases, except in early infancy, acupuncture, or the use of a fine trocar, often fails to cure. The walls of the cysts are usually thin, and collapse so much when their contents are withdrawn that the injection of a fluid is uncertain. The end of the canula may be outside the cyst, and the iodine solution be consequently injected into the connective tissue at its exterior. In such cases the following is a reliable method of treatment: "The cyst being well isolated, made tense, and brought near the surface, I pass through its centre a stout needle, armed with silk, and leave the threads hanging. The fluid quickly oozes away, especially if a little traction be made on the threads. I then, at one opening, wet the threads with iodine liniment (liniment because the quantity required is limited) and draw the threads so as to leave moistened portions within the cyst. A little gentle friction will help to spread the iodine thoroughly over the lining membrane of the cavity. An hour later freshly moistened portions may again be drawn through, if the cyst be large, or if other methods of treatment have failed. On the other hand, in a very small cyst a single thread, moistened and kept in one hour, will suffice.

"Another class of cases are those of simple vaginal hydrocele in which the injection of iodine and other ordinary methods of treatment are unsuccessful. An interesting case will best convey what I wish to say. A young man had a moderate-sized hydrocele. Trocar puncture and acupuncture repeated a few times failed, and consequently iodine tincture (undiluted) was injected. In a few weeks the collection had reached its old size.

A silver-wire seton was then put in ; while in the cyst remained empty, but its removal was followed by reappearance of the fluid. I then, at three o'clock passed through the cavity a double silk thread at two spots. In a few minutes, when all the fluid oozed out, I drew the threads, moistened with iodine linament, into the serous cavity. I directed him to repeat the process in in hour. He was so anxious to get well—he was shortly to be married—that he moistened the thread four times in six hours. At midnight the effects had become so sharp that he was glad to remove the threads as he had been directed. He remained at home one day only, and was shortly and permanently well.

"I venture to believe that no kind of hydrocele will resist this method of applying iodine, and consequently that the setting up of suppuration, even as a last resort, can rarely be necessary."

---

*Some Health Principles.*—No small share of disease and suffering is owing to the error of one man making another's experience a guide for himself in matters pertaining to bodily health.

Nothing can be more true, than that one may do with impunity what would kill another. We know a lady who will instantly take cold if she passes across a room or hall which has just been washed. The life of such a person would seem to hang on a very uncertain tenure. But, like a true philosopher, having found that passing through a recently washed room gives her cold, she simply avoids it; and now, at the full age of three score years and ten, scarcely misses a meal from sickness in a whole year.

When a man finds out that his constitution is a frail one, his wisest plan is to study its infirmities, to find out its weak points, and like a beleagured general, the winner of a hundred victories, be always on his guard as to those weak points. An old hat is never made better by being banged about, while by care, it mav be made to look *respectable* for years longer. A worn-out horse obtains no reinvigoration by hard usage. A man's body, whether frail or strong, is made capable of greater endurance by being well watched over. And take our word for it : *The best way to harden the constitution is to take care of it.*

That the popular sentiment should prevail that the human constitution is hardened by exposure, when there is nothing like it in the whole range of animated nature, must be classed among the unaccountabilities.

Some men thrive in spite of a given evil habit. This arises from two distinct causes, a vigorous constitution, or from the nature of the human body to adapt itself to the evils of life.—*Med. Brief.*

*Treatment of Cysts.*—Simple puncture should be made only for diagnosis. Every genuine cyst must be injected with iodine. One should use a trochar sufficiently large, so that small masses may come out. If the cystic fluid contains much sediment, or many granular pieces which have broken off, Lucke considers it very important to wash out the cyst with warm water before injecting the iodine (ten to twenty grammes according to the size of the cyst). The puncture is carefully closed with sticking-plaster or collodion. In many cases cure takes place. Some operators wishing to encourage suppuration, inject a dilute solution of chloride of iron, and leave in the canula (Mackenzie).

Incision of the cyst is well esteemed by Lucke. After an incision has been made down to the cyst wall, this should be stitched to the skin on either side, and the cut into the cyst made between the stitches. If the operation be done in this manner, the bleeding will be less. After the contents of the cyst are evacuated, bleeding from the incision in the cyst wall is best stopped by additional sutures, but bleeding from the interior of the sac must be stopped by plugging with lint. Cure takes place by suppuration, and one must take care that the external opening does not close too soon. Secondary hœmorrhage is the accident most to be feared as suppuration goes on. The vessels cannot usually be found, and plugging up the cavity must be resorted to. Purulent infiltration and septicæmia are also possible, usually, however, only in conjunction with hemorrhage in persons already enfeebled. In other respects the results of this mode of treatment are very brilliant. Sometimes after the cyst is laid bare the connections with the surrounding tissues may be so loose that it will be better to practice extirpation.—*Exchange.*

---

*Treatment of Children in Egypt.*—A correspondent of the London *Times*, writing from Egypt, says:

The philosopher who deemed him happiest who has the fewest wants ought to have an Egyptian fellah. He is sometimes even born in the fields. The women work up to the day of their confinement. They lie up one day, and are out again the next, and the baby is laid near them in the field on a bit of sacking. Ignorance and poverty lead to other sad consequences. Premature old age comes on at 40, and the population is kept down by a terrible infant mortality. Out of the 140,000 annual deaths, 80,000 are of infant children. It has been calculated that three out of every five that are born die before the age of two. For those that survive, an old Egyptian custom that is still practiced is symbolical of their future. The child is put into a sieve and rolled about to the beating of drums. "It is in order to harden him," say the people.—*Pacific Med. & Surg. Journal.*

---

*Vegetable Diet in Relation to Longevity.*—The vegetarians throughout the world have been brought to grief by the report of Mr. Howlett, Office of health for Bombay, who states that while the general mortality in the 816,562 inhabitants of Bombay amounts to 1.89 per cent., among the 61,294 non-flesh-eating casts belonging to the higher grades of society, the mortality amounts to 2.05 per cent. That abstinence from flesh should increase the death-rate by 16 per cent. presents a curious puzzle for the vegetarians, only equalled by the further fact that the Burmese, whose religion makes them reverence all life. are remarkable for the number of murders they commit.—*Pacific Med. and Surg. Journal.*

---

*An Extraordinary Story.*—A *post-mortem* examination made upon the body of a lunatic patient in the Prestwich Asylum, led to the discovery of no fewer than 1841 articles in his inside, viz : 1639 shoemaker's sparables, six four-inch cut nails, nineteen three-inch cut nails, eight two and a half-inch cut nails, eighteen two-inch cut nails, forty half-inch cut nails, seven three-eighth-inch cut nails, thirty-nine tacks, five brass nails, nine brass buttons, twenty pieces of buckles, one pin, fourteen bits of glass, ten small pebbles, three pieces of string, one piece of leather three inches long, one piece of lead four inches long, and one American pegging awl ; the total eleven pounds ten ounces.—[*Press & Circular.*]

---

*Treatment of Stammering.*—One of the dailies contains a letter, which appears trustworthy, written by a gentleman who stammered from childhood almost up to manhood, and who gives a very simple remedy for the misfortune : "Go into a room where you will be quiet and alone, get some book that will interest but not excite you, sit down and read two hours aloud, to yourself, keeping your teeth together. Do the same every two or three days, or once a week if very tiresome, always taking care to read slowly and distinctly, moving the lips but not the teeth. Then, when conversing with others, try to speak as slowly and distinctly as possible. and make up your mind that you will not stammer. Well, I tried his remedy, not having much faith in it, I must confess, but willing to do almost anything to cure myself of such an annoying difficulty. I read for two hours aloud, with my teeth together. The first result was to make my tongue and jaws ache, that is, while I was reading, and the next to make me feel as if something had loosened my talking apparatus, for I could speak with less difficulty immediately. The change was so great that every one who knew me remarked it. I repeated the remedy every five or six days for a month, and then at longer intervals, until cured."—*Medical Brief.*

---

*Hiccough.*—Dr. Simon reports that a patient who had hiccoughed for twenty-six hours, had been subjected to various treatments without success, was relieved almost instantaneously by inhaling three drops of the amyl nitrite.—*Chicago Med. Jour. and Exam.*

---

*Nitrite of Amly.*—Dr. Squibb says the practice of administering the nitrite of amly is drifting into the inhalation of small quantities from a small vial, the mouth of which is applied to one nostril; one to three full inspirations being sufficient. This small vial may be carried by asthmatics and others in the vest-pocket; should be tightly corked, corks are rapidly rendered friable by the nitrite. This method is convenient and economical.

Dr. Squibb has information that the nitrite is being largely employed in the asylums of this country for the treatment of the epileptic insane. Dr. Kempster says that his epileptic wards have been revolutionized: They are less noisy, the seizures becoming comparatively rare, by reason of the power of the drug to anticipate and prevent them, where the aura is distinct, or to arrest them where there is no appreciable aura. The nurses are instructed in its use. As an explanation of the alleged curative effect of the remedy, Dr Squibb suggested that it may cure by breaking up the epileptic habit, as appears in the case of other affections showing a periodic tendency.—*Med. Brief.*

---

*Cephalalgia.*—D. Lithgow states that he has been in the habit, since 1869, of using nitrate of amyl in nervous headaches, as recommended by Sir J. V. Simpson; and where due care was taken in the selection of cases, he never knew it to fail to produce entire and almost immediate relief.—*Lancet.*

---

*Carbolic-Acid sprays in throat affections* is highly recommended by James Cuthill, M. D., in *The British Medical Journal.* The writer says that for more than twelve months he has treated all his cases of diphtheria and of ulceration of the tonsils and fauces by means of carbolic acid spray, except that in some of the more severe instances the solid nitrate of silver has also been applied. Excellent effects have likewise been attained in relaxation of the uvula and other non-ulcerative conditions, as well as in scarlatinal sore throats. Among the beneficial effects of this method of treatment are the removal of the sickening fetor of the breath and the speedy restoration of the power of deglutition. The strength of the solution may be from one in forty to one in twenty. No spatula is required, the stream of spray being merely directed over the dorsum of the tongue.

---

*Natural Ink.*—In Algeria there is a river of genuine ink. It is formed by the union of two streams, one coming from a region of ferruginous soil, the other draining a peat swamp The water of the former is strongly impregnated with iron. that of the latter with tannic acid. When the two waters mingle, the acid of the one unites with the iron of the other, forming a true ink.—*Med. Brief.*

---

*To Open an Abscess Without Pain.*—Dr. Bergonzini reports in the *Revista Clin. de Bologna* that if the following solution be applied to the skin over an abscess for three to five minutes an incision may be made into it without pain : Carbolic acid, two parts ; glycerine, one part.

---

*How to Cure a Cold in the Head.*—Dr. Ferrier, being dissatisfied with the usual formula for treating a cold in the head, namely, "sodorifics and lying in bed," has made a trial of various substances, to be used in the form of snuffs at the beginning of an attack of nasal catarrah. He finds the following prescription very efficacious :

| | | |
|---|---|---|
| R. | Muriate of morphia, - - | 2 grains. |
| | Acacia powder, - - - - | 2 drachms. |
| | Subnitrate of bismuth, - - | 2 drachms. |

Of the powder one-quarter to one-half may be taken as snuff in the twenty-four hours.—*Med Brief.*

---

*Eye-sight.*—There are many persons who are greatly annoyed with weak eyes. This results, sometimes, from using the eyes in too great a glare of light; at others, from using them by artificial light, or in the "dusk of the evening;" that is by twilight. To illustrate, in part: Our office has two large windows; twelve sets of slats cover them. If all the slats are open, our eyes begin to water in five minutes, and we commence a winking and a blinking, which has to be kept up all the time, to keep the sight clear. But if we close all the slats but one, and that an upper one, so as to throw the light down on the page, and from behind, we can write for hours without inconvenience —or "specs." We conclude, therefore, that we have been using eleven times more light than was necessary. This simple fact may be of real practical use to multitudes. Too much light injures the eyes, as certainly as too little. We found this out today, after a variety of experiments within two months; thus we are living to learn, with a view of learning to live—for a long time. The amount of relief afforded, in any particular case, can only be determined by experiment.—*Hall's Journal of Health.*

---

*Wounds.*—The secret of success in the treatment of wounds, depends upon the removal of all foreign bodies; upon arresting of hemorrhage; upon bringing the edges evenly and neatly together; in joining the edges with metallic sutures, and in keeping them there with adhesive strips and bandages; and lastly in excluding atmospheric air. In all cases, sprinkling over the wound pulverized borax is to be recommended as a mild, efficient antiseptic.—*Exchange.*

---

*Nervous Headache and Neuralgia.*—

| | |
|---|---|
| Quinine, - - - - - | 24 grains. |
| Tannin, - - - - - - | 18 grains. |
| Ext. aconite, - - - - | 6 grains. |

Mix and divide in 24 pills.

Sig—One pill night and morning.—*Med. Brief.*

---

*Soft Hands.*—To preserve the smoothness and softness of the hands keep a small bottle of glycerine near the place where you habitually wash them, and whenever you have finished washing, and before wiping them, put one or two drops of the glycerine on the wet palm and rub the hands thoroughly with it as if it were soap, then dry lightly with a towel. Household work and bad weather will not prevent your skin from being smooth and soft if this plan of using glycerine is followed.—*Med. Brief.*

